# DL-ICE as a bridge to allogeneic transplantation in relapsed/refractory PTCL: survival outcomes and prognostic factors

**DOI:** 10.3389/fonc.2024.1461268

**Published:** 2024-12-09

**Authors:** Tong-Yoon Kim, Tae-Jung Kim, Eun Ji Han, Gi June Min, Seok-Goo Cho, Youngwoo Jeon

**Affiliations:** ^1^ Department of Hematology, Yeouido St. Mary’s Hospital, College of Medicine, The Catholic University of Korea, Seoul, Republic of Korea; ^2^ Lymphoma and Cell Therapy Research Center, Yeouido St. Mary’s Hospital, College of Medicine, The Catholic University of Korea, Seoul, Republic of Korea; ^3^ Department of Hospital Pathology, Yeouido St. Mary’s Hospital, College of Medicine, The Catholic University of Korea, Seoul, Republic of Korea; ^4^ Division of Nuclear Medicine, Department of Radiology, Yeouido St. Mary’s Hospital, College of Medicine, The Catholic University of Korea, Seoul, Republic of Korea; ^5^ Department of Hematology, Seoul St. Mary’s Hospital, College of Medicine, The Catholic University of Korea, Seoul, Republic of Korea

**Keywords:** L-asparaginase, non-Hodgkin lymphomas, relapsed/refractory, stem cell transplantation, T-cell lymphomas

## Abstract

**Introduction:**

Peripheral T-cell lymphomas (PTCLs) have poor outcomes in the relapsed/refractory (R/R) setting. In this study, we evaluated the efficacy of dexamethasone, L-asparaginase, ifosfamide, carboplatin, and etoposide (DL-ICE) chemotherapy followed by allogeneic hematopoietic stem cell transplantation (allo-HSCT) in patients with R/R PTCLs.

**Methods:**

We retrospectively analyzed 80 adult patients with R/R PTCLs treated with DL-ICE chemotherapy between September 2009 and March 2023. Patients achieving complete or partial remission were eligible for consolidative allo-HSCT. Overall survival (OS) and progression-free survival (PFS) were evaluated.

**Results:**

The overall response rate to DL-ICE was 37.5%, with 30% achieving complete remission (CR). With a median follow-up of 96.4 months, the median OS and PFS were 8.9 and 3.8 months, respectively. Seventeen patients (21%) underwent allo-HSCT, including 11 with non-CR status. The 5-year OS was significantly higher in the allo-HSCT group compared to that in the group with chemotherapy alone (64.7% vs 18.3%, p <0.001). Multivariate analysis identified advanced stage, EBV viremia, and non-CR status as poor prognostic factors.

**Discussion:**

DL-ICE chemotherapy demonstrated modest activity in R/R PTCLs. Consolidation with allo-HSCT, even in patients who do not achieve CR, resulted in long-term survival in a subset of patients. Early consideration of allo-HSCT may improve outcomes for patients with R/R PTCLs.

## 1 Introduction

Peripheral T-cell lymphomas (PTCLs) are a heterogeneous group of aggressive non-Hodgkin lymphomas with poor prognosis, particularly in the relapsed/refractory (R/R) setting (1). Despite advances in front-line therapy, 30–40% of patients experience relapse or refractory disease (2). The prognosis for R/R PTCLs remains poor, with a median overall survival (OS) of <1 year when treated with conventional salvage chemotherapy alone (3). High-dose chemotherapy followed by autologous stem cell transplantation (auto-SCT) has been the standard approach for chemosensitive relapse. However, outcomes remain poor, with 3-year progression-free survival (PFS) rates of only 20–30% (4). This has led to increased interest in allogeneic hematopoietic stem cell transplantation (allo-HSCT) as a potentially curative option for R/R PTCLs (5). L-asparaginase has shown promising activity in extranodal NK/T-cell lymphoma (6). Interestingly, some reports have highlighted its effectiveness against PTCL NOS (7), leading to its incorporation into salvage regimens for other T-cell lymphomas. The combination of dexamethasone, L-asparaginase, ifosfamide, carboplatin, and etoposide (DL-ICE) represents a novel salvage approach for R/R PTCLs, building upon established ICE regimens (8). Recent studies have highlighted the potential benefit of allo-HSCT in R/R PTCLs, even in patients who did not achieve complete remission (CR) (9). However, the optimal timing and patient selection for allo-HSCT remain controversial. Additionally, the role of Epstein–Barr virus (EBV) in prognosis and treatment response has recently gained attention^3^. This study evaluated the efficacy and safety of DL-ICE chemotherapy followed by allo-HSCT in patients with R/R PTCLs. We hypothesized that this approach would improve outcomes compared with historical data with conventional salvage therapy alone and sought to identify prognostic factors that could guide treatment decisions.

## 2 Methods

### 2.1 Patient Selection

This retrospective study included adult patients (≥18 years) with histologically confirmed R/R PTCLs treated at Seoul St. Mary’s Hospital and Yeouido St. Mary’s Hospital between September 2009 and March 2023. Eligible PTCL subtypes included PTCL not otherwise specified (PTCL-NOS), angioimmunoblastic T-cell lymphoma (AITL), anaplastic large cell lymphoma, extranodal NK/T-cell lymphoma (ENKTL), and other rare subtypes. Patients must have received at least one prior line of therapy and to have measurable disease at the time of relapse. Candidates whose skin allergic test were positivity for L-asparaginase were excluded from the study. The study was approved by the Institutional Review Board of the Catholic Medical Center (XC23RADI0045). Patient consent was not needed because of the retrospective nature of the study.

### 2.2 Treatment Protocol

All patients received DL-ICE chemotherapy consisting of:

- Dexamethasone 20 mg/day on days 1–4
- L-asparaginase 4,000 IU/m^2^ on days 1–4
- Ifosfamide 5 g/m^2^ over 24 h on day 1
- Carboplatin AUC 5 on day 1
- Etoposide 100 mg/m^2^ on days 1–3

Cycles were repeated every 21–28 days for a maximum of four cycles. Granulocyte colony-stimulating factor support was administered according to institutional guidelines. All patients were hospitalized until chemotherapy was completed to monitor for side effects.

Patients achieving CR or partial remission (PR) following DL-ICE were considered for consolidative allo-HSCT. Lack of available donors or patients’ disagreement with allo-HSCT were reasons for allocation to autologous HSCT (auto-HSCT). For auto-HSCT, patients received G-CSF (filgrastim, 10 µg/kg) for mobilization 48 h after the last cycle of DL-ICE infusion. Apheresis was performed when leukocyte and peripheral CD34 counts were elevated. In ASCT, patients were treated with reduced-intensity conditioning using the BuMelTT protocol, which consisted of busulfan (2.4 mg/kg) on days -8 to -6, melphalan (40 mg/m²) on days -5 to -4, and thiotepa on days -3 to -2, as previously described (10).

In the allo-HSCT group, patients received a fludarabine, melphalan, and total body irradiation (FMT) regimen comprising 30 mg/m² fludarabine on days -9 to -4, 70 mg/m² melphalan on day -3, and total body irradiation of 400 cGy on days -2 to -1. Anti-thymocyte globulin (rabbit ATG, 2.5–5.0 mg/kg; Genzyme Transplant, Cambridge, MA, USA) was administered for graft-versus-host disease (GVHD) prophylaxis in mismatched grafts on days -2 to -1. After transplantation, cyclosporine was used for full matched sibling donors, while tacrolimus was administered for other types of donors to control GVHD. Methotrexate was infused on days 1, 3, 6, and 11 (5 mg/m² for fully matched sibling donors and 10 mg/m² for other donors) (11).

### 2.3 Response Assessment and Statistical Analysis

Treatment response was assessed using computed tomography (CT) and positron emission tomography after two cycles and at the end of treatment. In patients from the auto- and allo-HSCT groups, assessment was performed before transplantation, utilizing the Lugano classification (12). CR is defined as the disappearance of target lymphoma lesions on CT, with normalization of 18F-fluorodeoxyglucose PET uptake in all involved sites (Deauville score of 1–3). PR was defined as a ≥50% regression in the target mass (Deauville score of 4–5). Progressive disease (PD) was characterized by a >25% increase in the target mass or the presence of new lesions (13).

OS was calculated from the start of DL-ICE until death from any cause or the last follow-up. PFS was calculated from the start of DL-ICE until disease progression, death, or the last follow-up. The presence of EBV *in situ* hybridization was detected using EBV-encoded small RNA oligonucleotides added to formalin-fixed paraffin-embedded samples, following the Inform EBER Probe Assay Protocol (Ventana Medical Systems Inc., Oro Valley, AZ, USA). EBV DNA load in peripheral blood was measured using real-time quantitative polymerase chain reaction at baseline and during follow-up. EBV viremia was defined >500 copies/mL. Survival curves were estimated using the Kaplan–Meier method and compared using the log-rank test. Univariate and multivariate Cox proportional hazards models were employed to identify prognostic factors. Statistical analyses were performed using R version 4.0.2 (R Foundation for Statistical Computing, Vienna, 2022).

## 3 Results

### 3.1 Patient Characteristics

In total, 80 patients with R/R PTCLs were included in the analysis. The median age was 48.5 years (range: 20–65), and 56% was male. PTCL subtypes included PTCL-NOS (41%), ENKTL (31%), AITL (13%), and others (15%). Most patients had advanced-stage disease (78%) and elevated lactate dehydrogenase (LDH) (73%) at relapse. Approximately 39% of the patients had primary refractory disease, while 61% had relapsed after the initial response. Moreover, 50% had detectable EBV viremia at baseline (**Table 1**).

**Table 1:** Patients’ characteristics.

		ALLO, N=17	AUTO, N=9	CTX, N=54	ALLO vs. AUTO, P	AUTO vs. CTX, P	ALLO vs. CTX, P
Age at treatment failure, years, median (range)	48.5 (20–65)	43.0 (22–65)	47.0 (20–65)	50.0 (22–65)	0.467	0.366	0.07
Sex, N, (%)					0.999	0.999	0.858
Female	34 (43.6)	8 (47.1)	4 (44.4)	22 (40.7)			
Male	44 (56.4)	9 (52.9)	5 (55.6)	32 (59.3)			
Diagnosis, N, (%)					0.504	0.418	0.558
PTCL	33 (41.2)	7 (41.2)	2 (22.2)	24 (44.4)			
ENKT	25 (31.2)	5 (29.4)	3 (33.3)	17 (31.5)			
AITL	10 (12.5)	2 (11.8)	3 (33.3)	5 (9.3)			
ALCL	7 (8.8)	1 (5.9)	1 (11.1)	5 (9.3)			
CTCL	3 (3.8)	2 (11.8)	0 (0.0)	1 (1.9)			
MEITLE	2 (2.5)	0 (0.0)	0 (0.0)	2 (3.7)			
Ann Arbor Stage, N, (%)					0.739	0.144	0.422
I–II	18 (22.5)	5 (29.4)	4 (44.4)	9 (16.7)			
III–IV	62 (77.5)	12 (70.6)	5 (55.6)	45 (83.3)			
ECOG, N, (%)					NA	0.068	0.008
0–1	60 (75.0)	17 (100.0)	9 (100.0)	34 (63.0)			
≥2	20 (25.0)	0 (0.0)	0 (0.0)	20 (37.0)			
Serum LDH level > upper normal range, N, (%)					0.514	0.459	> 0.999
Negativity	22 (27.5)	4 (23.5)	4 (44.4)	14 (25.9)			
Positivity	58 (72.5)	13 (76.5)	5 (55.6)	40 (74.1)			
Bone marrow involvement, N, (%)					>0.999	0.491	0.58
Negativity	51 (63.7)	12 (70.6)	7 (77.8)	32 (59.3)			
Positivity	29 (36.2)	5 (29.4)	2 (22.2)	22 (40.7)			
EBV (ISH), N, (%)					0.797	>0.999	0.651
Negativity	31 (38.8)	8 (47.1)	3 (33.3)	20 (37)			
Positivity	49 (61.3)	9 (52.9)	6 (66.7)	34 (67)			
EBV (PB) >500 copies, N, (%)					0.514	0.64	0.022
Negativity	40 (50.0)	13 (76.5)	5 (55.6)	22 (40.7)			
Positivity	40 (50.0)	4 (23.5)	4 (44.4)	32 (59.3)			
Refractory, N, (%)					> 0.999	0.958	0.907
Relapsed	31 (38.8)	6 (35.3)	3 (33.3)	22 (40.7)			
Refractory	49 (61.3)	11 (64.7)	6 (66.7)	32 (59.3)			
Response, N, (%)					0.028	< 0.001	0.267
CR	24 (30.0)	6 (35.3)	8 (88.9)	10 (18.5)			
Non-CR	56 (70.0)	11 (64.7)	1 (11.1)	44 (81.5)			
Frontline chemotherapy, N, (%)					0.071	0.179	0.21
CHOEP	2 (2.5)	1 (5.9)	0 (0.0)	1 (1.9)			
CHOP	30 (37.5)	3 (17.6)	5 (55.6)	22 (40.7)			
EPOCH	1 (1.2)	1 (5.9)	0 (0.0)	0 (0.0)			
ESHAP	2 (2.5)	0 (0.0)	1 (11.1)	1 (1.9)			
ProMACE	43 (53.8)	12 (70.6)	2 (22.2)	29 (53.7)			
SMILE	2 (2.5)	0 (0.0)	1 (11.1)	1 (1.9)			
Chemo therapy line, N, (%)					> 0.999	0.904	> 0.999
2	76 (95.0)	16 (94.1)	8 (88.9)	52 (96.3)			
3	4 (5.0)	1 (5.9)	1 (11.1)	2 (3.7)			

ALLO, allogeneic hematopoietic stem cell transplantation, AUTO, autologous hematopoietic stem cell transplantation; CTX, chemotherapy only; vs., versus; N, number; P, p-value; ENKT, extranodal NK/T-cell lymphoma; PTCL, peripheral T-cell lymphoma, not otherwise specified; AITL, angioimmuoblastic T-cell lymphoma; CTCL, cutaneous T-cell lymphoma; MEITLE, monomorphic epitheliotropic intestinal T-cell lymphoma; ECOG, Eastern Cooperative Oncology Group performance status; LDH, lactate dehydrogenase; EBV (ISH), Epstein–Barr virus *in situ* hybridization histology; EBV (PB), Epstein–Barr virus in peripheral blood; CR, complete remission; Non-CR, non-complete remission; CHOEP, cyclophosphamide, doxorubicin, etoposide, vincristine and prednisone; CHOP, cyclophosphamide, doxorubicin, vincristine and prednisone; EPOCH, etoposide, prednisone, vincristine, cyclophosphamide, and doxorubicin; ESHAP, etoposide, solumedrol, high-dose cytarabine, and cisplatin; ProMACE, cyclophosphamide, doxorubicin, etoposide, bleomycin, vincristine, methotrexate and prednisone; SMILE, dexamethasone, methotrexate, ifosfamide, L-asparaginase, etoposide. NA, "Not applicable".

### 3.2 Response to DL-ICE and Survival Outcomes

**Figure 1:**
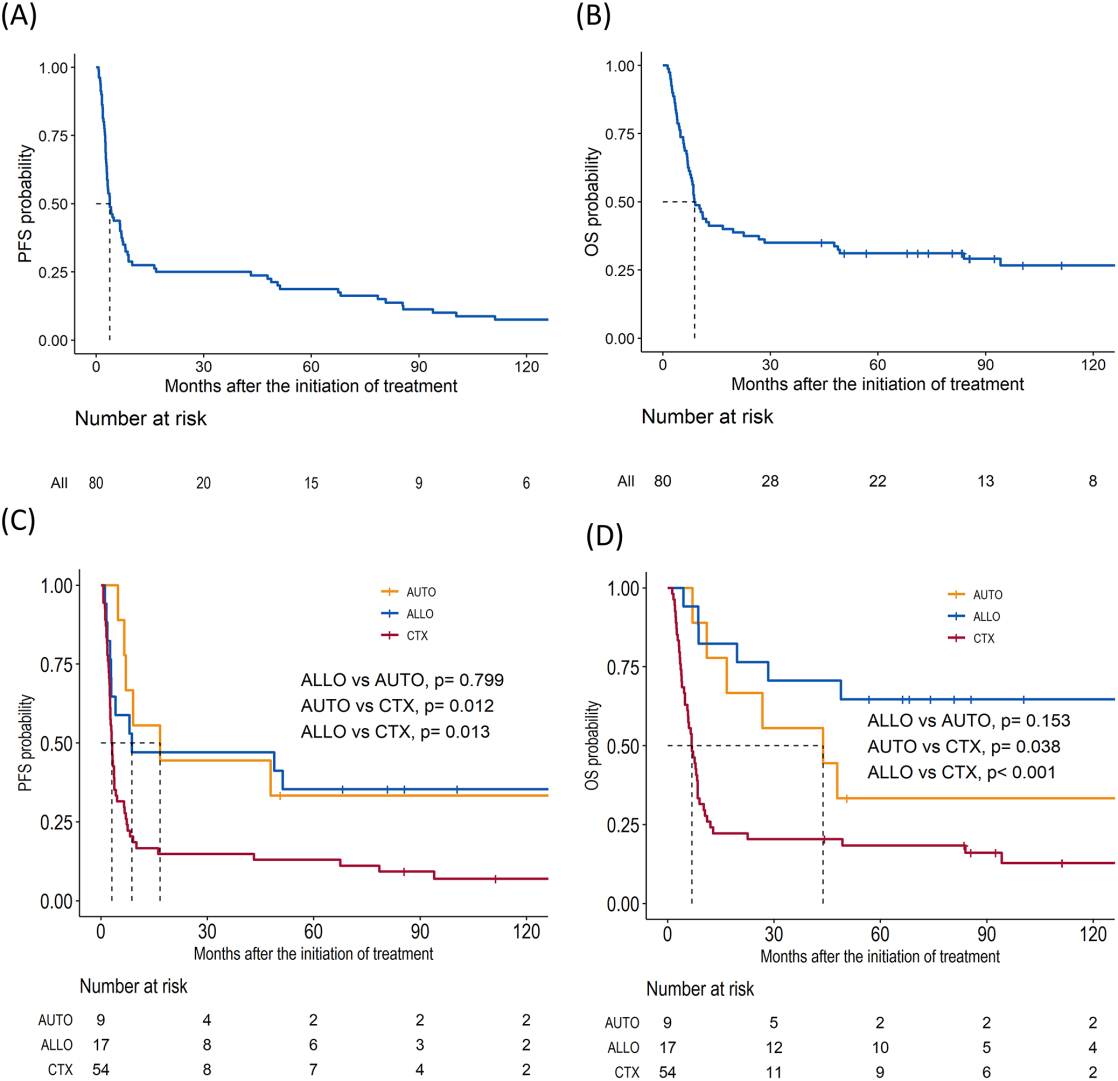
Survival outcomes for patients with relapsed/refractory peripheral T-cell lymphomas who underwent salvage chemotherapy. **(A)** Progression-free survival (PFS) in total cohort. **(B)** Overall survival (OS). **(C)** PFS of allogeneic hematopoietic stem cell transplantation (ALLO), autologous hematopoietic stem cell transplantation (AUTO), and chemotherapy only group (CTX). **(D)** OS of ALLO, AUTO, and CTX.

**Figure 2:**
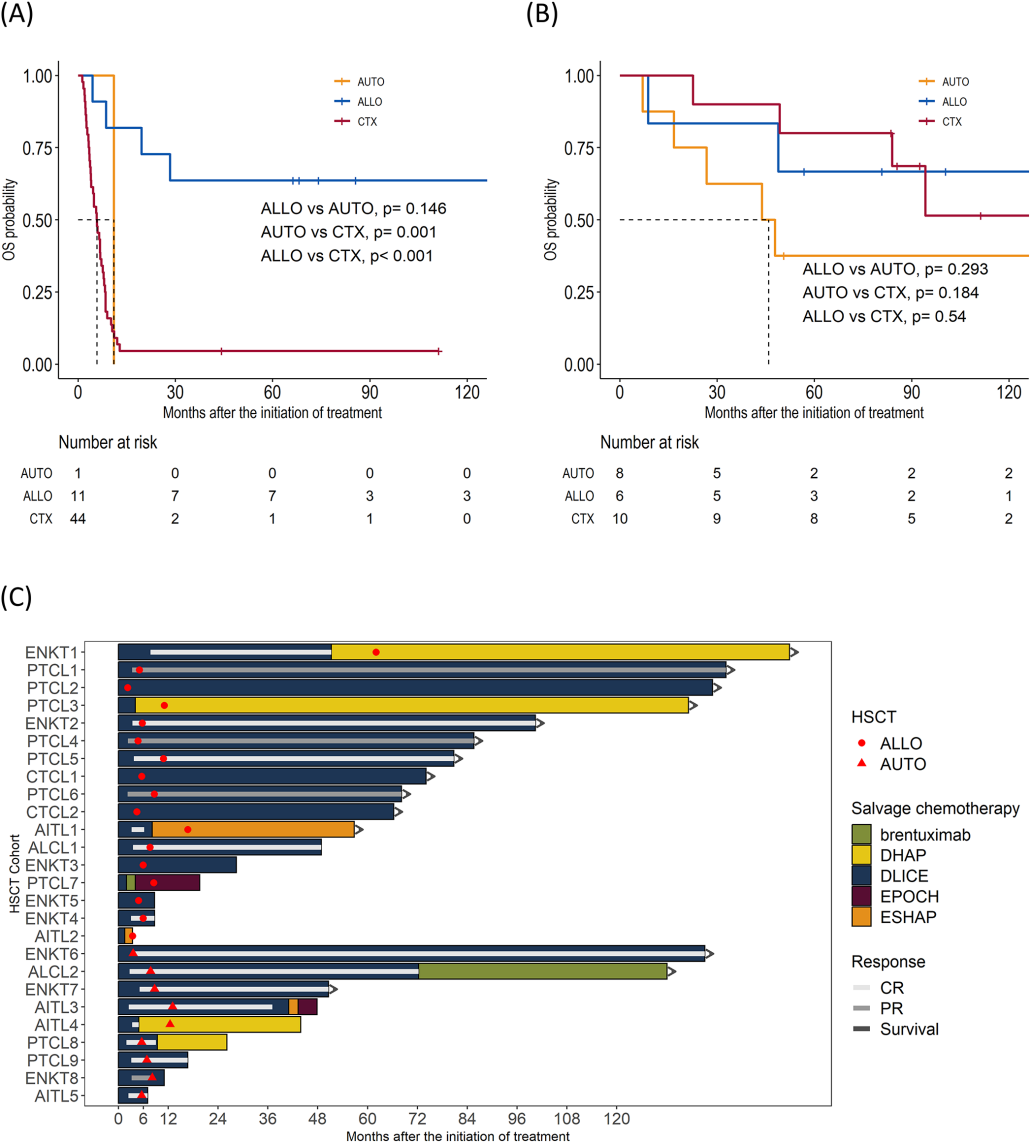
Impact on survival outcomes of hematopoietic stem cell transplantation (HSCT) by subgroups. **(A)** Kaplan–Meier curve of overall survival (OS) in patients with less than complete remission (CR) before HSCT **(B)** OS in CR. **(C)** Swimmers plot of patients who underwent HSCT.

The overall response rate to DL-ICE chemotherapy was 37.5%, including 30% CR and 7.5% PR. These results are comparable with other salvage regimens, such as DHAP (dexamethasone, high-dose cytarabine, and cisplatin) and GDP (gemcitabine, dexamethasone, and cisplatin), which have reported overall response rates of 30–50% in R/R PTCLs (14, 15). With a median follow-up of 96.4 months, the median OS and PFS for the entire cohort were 8.9 (95% CI: 7.9–22.6) months and 3.8 (95% CI: 3.0–7.0) months, respectively, similar to those reported in the SCHOLAR-1 study, which found a median OS of 6.3 months in refractory aggressive non-Hodgkin lymphomas (14) (**Figures 1A, B**). Seventeen patients (21%) proceeded to allo-HSCT, including six in CR and 11 with less than CR. Median PFS for in the allo-HSCT group were 8.7 months. The 5-year OS rate was significantly higher in the allo-HSCT group than in those receiving chemotherapy alone (64.7% vs. 18.3%, p <0.001) Nine patients were allocated to auto-HSCT, including eight patients who achieved CR and one patient with less than CR. The median PFS was 16.7 months, and the 5-year OS rate was 33.3% (**Figures 1C, D**). Notably, patients undergoing allo-HSCT with less than CR achieved a 5-year OS of 63.6%, is consistent with recent studies suggesting that allo-HSCT can overcome chemoresistance in PTCLs through a graft-versus-lymphoma effect (**Figures 2A, B**) (16). Detailed treatment courses in patients underwent HSCT described in **Figure 2C**.

### 3.3 Prognostic Factors

In multivariate analysis, advanced Ann Arbor stage (hazard ratio [HR]: 3.33, 95% confidence interval [CI]: 1.45–7.63, p=0.004), EBV viremia >500 copies/mL (HR: 3.50, 95% CI: 1.86–6.59, p <0.001), and failure to achieve CR with DL-ICE (HR: 3.27, 95% CI: 1.56–6.84, p=0.002) were independently associated with inferior OS (**Table 2**).

**Table 2:** Univariate and multivariate analyses of risk factors in all patients.

Variable	HR, 95% CI	p-value	HR, 95% CI	p-value
Age at treatment failure >60 vs. ≤60 years	1.27 (0.64–2.51)	0.498		
Male vs. Female	1.03 (0.61, 1.73)	0.919		
ENKT vs. The other	0.85 (0.48–1.48)	0.563		
Ann Arbor Stage III–IV vs. I–II	3.43 (1.60–7.33)	0.001	3.33 (1.45–7.63)	0.004
ECOG >2 vs. ≤ 2	2.33 (1.31–4.16)	0.004	1.27 (0.65–2.47)	0.482
Serum LDH level > UNL vs ≤ UNL	0.96 (0.54–1.69)	0.886		
Bone marrow involvement, positivity vs negativity	2.12 (1.25–3.60)	0.005	1.28 (0.73–2.22)	0.387
EBV (ISH) positivity vs negativity	1.50 (0.86–2.59)	0.150		
EBV (PB) >500 copies vs. ≤500	2.66 (1.55–4.57)	<0.001	3.5 (1.86–6.59)	<0.001
Refractory vs. relapse	2.09 (1.19–3.68)	0.01	1.39 (0.74–2.63)	0.308
Response, Non-CR vs. CR	3.74 (1.91–7.31)	<0.001	3.27 (1.56–6.84)	0.002
Chemo therapy three lines vs. two lines	1.07 (0.33–3.42)	0.914		

HR, hazard ratio; CI, confidence interval; vs., versus; ENKT, Extranodal NK/T-cell lymphoma; ECOG, Eastern Cooperative Oncology Group performance status; LDH, lactate dehydrogenase; UNL, upper normal limit; EBV (ISH), Epstein–Barr virus *in situ* hybridization histology; EBV (PB), Epstein–Barr virus in peripheral blood; CR, complete remission; Non-CR, non-complete remission.

### 3.4 Toxicity in the Salvage Chemotherapy and Post-Allo-HSCT Complications

All patients were admitted until the completion of chemotherapy to monitor for L-asparaginase-related toxicity. The most common grade 3–4 toxicities with DL-ICE were hematologic, including neutropenia (69%), thrombocytopenia (55%), and anemia (25%). Febrile neutropenia occurred in 16% of patients. These rates are comparable with those reported with other intensive salvage regimens (17). Non-hematologic toxicities were generally mild, with grade 3–4 events, including hepatitis (3%) and thrombosis (1%) (**Table 3**). The incorporation of L-asparaginase did not appear to significantly increase toxicity compared to standard ICE chemotherapy (8). Among allo-HSCT recipients, acute GVHD grades II–IV occurred in 41% of patients, while chronic GVHD developed in 35%. There were three cases of transplant-related mortality. These complication rates are consistent with those reported in other studies of allo-HSCT for PTCLs (16).

**Table 3:** Treatment-related adverse events.

Adverse event	Grade 1	Grade 2	Grade 3	Grade 4	Grades 3–4	Any grade
Non-hematologic						
hepatitis, N, (%)	0 (0)	8 (10)	2 (3)	0 (0)	2 (3)	10 (12)
Acute kidney injury, N, (%)	3 (4)	2 (3)	0 (0)	0 (0)	0 (0)	5 (6)
Pancreatitis, N, (%)	2 (3)	2 (3)	0 (0)	0 (0)	0 (0)	4 (5)
Thrombus, N, (%)	0 (0)	0 (0)	1 (1)	0 (0)	1 (1)	1 (1)
Hematologic						
Thrombocytopenia, N, (%)	0 (0)	5 (6)	20 (25)	24 (30)	44 (55)	49 (61)
Anemia, N, (%)	2 (3)	13 (16)	19 (24)	1 (1)	20 (25)	35 (44)
Neutropenia, N, (%)	0 (0)	0 (0)	5 (6)	50 (62)	55 (69)	55 (69)
Febrile neutropenia, N, (%)	0 (0)	0 (0)	3 (4)	10 (12)	13 (16)	13 (16)

N, Number.

## 4 Discussion

This study evaluated the efficacy of the DL-ICE chemotherapy regimen followed by allo-HSCT in patients with R/R PTCLs. The overall response rate of 37.5% to DL-ICE is modest but comparable to that of other salvage regimens in this setting (14, 15). Notably, the incorporation of L-asparaginase did not significantly increase toxicity compared with standard ICE chemotherapy, suggesting that this regimen is a viable option for patients who have failed previous platinum-based salvage therapy. However, L-asparaginase-related toxicities, such as pancreatitis, thromboembolism, and hepatitis, necessitate caution. Additionally, we excluded patients who tested positive for an allergic reaction to L-asparaginase, thereby reducing the risk of anaphylactic shock.

In comparison to previous reports, the PFS for patients receiving ICE therapy did not show a significant improvement, with our cohort demonstrating a PFS of 3.8 months compared to the reported range of 3.4–4.4 months. However, patients who underwent allo-HSCT exhibited a superior OS rate compared to the 1-year OS rates of 51.0–59.6% previously reported (18). The most striking finding of this study was the superior long-term survival observed in patients who proceeded to allo-HSCT, even among those with less than CR. The 5-year OS of 64.7% for transplanted patients compares favorably with historical data for conventional salvage approaches (3, 4). This supports the potential benefit of the graft-versus-lymphoma effect in T-cell malignancies, as previously suggested (16). Notably, 11 of the 17 transplanted patients had less than CR at the time of allo-HSCT, yet achieved a 5-year OS of 63.6%. This finding challenges the traditional paradigm that requires CR prior to transplantation and suggests that allo-HSCT should be considered earlier in the disease course for eligible patients with R/R PTCLs, rather than pursuing multiple lines of salvage therapy. This approach is further supported by recent studies indicating that an increased number of treatment lines prior to transplant is associated with worse outcomes (16).

The role of L-asparaginase in the DL-ICE regimen deserves special attention. While L-asparaginase has shown remarkable efficacy in extranodal NK/T-cell lymphoma, its utility in other PTCL subtypes remains limited. Our study suggests that incorporating L-asparaginase into the ICE backbone provides additional benefits without significantly increasing toxicity. This is particularly important given the limited treatment options for patients with R/R PTCLs. Future studies should consider a randomized comparison of ICE versus DL-ICE to definitively assess the contribution of L-asparaginase in this setting. The timing of allo-HSCT in the treatment course of R/R PTCLs remains to be addressed. Our data suggest that earlier referral for transplantation, even in patients who have not achieved CR, may be beneficial (19). However, the decision to proceed with transplant must be balanced against the risks of transplant-related mortality and GVHD.

In our cohort, the rates of acute and chronic GVHD were considerable but manageable and in line with previous reports (20). Strategies to mitigate GVHD while preserving the graft-versus-lymphoma effect, such as post-transplant cyclophosphamide, which has shown tolerable outcomes in patients with PTCLs, warrant further investigation in this patient population (21). We recommend considering allo-HSCT over auto-HSCT due to the low CR rates and the short PFS observed in our cohort. Waiting for CR appears less effective in the R/R setting compared to the chemotherapy-naive context. However, in situations where donor availability is limited, a careful application of auto-HSCT may still offer survival benefits in patients who achieve CR.

The identification of advanced stage and chemoresistance as poor prognostic factors is consistent with previous findings in PTCLs (22). Furthermore, the strong prognostic impact of EBV viremia, which is a potential biomarker for PTCLs and other lymphomas (23–25), underscores the possible role of EBV-directed therapies, such as rituximab or EBV-specific cytotoxic T lymphocytes, in the management of EBV-positive PTCLs (26).

While rituximab has been utilized in the treatment of EBV-positive B-cell lymphomas, its role in T-cell lymphomas remains inadequately defined. Emerging therapies, such as EBV-specific cytotoxic T lymphocytes or small molecule inhibitors targeting EBV-driven pathways, may provide new avenues for treatment (27). Additionally, employing EBV DNA load as a biomarker for assessing treatment response and early relapse detection could potentially inform treatment decisions and enhance patient outcomes.

Notably, although our results with allo-HSCT are promising, not all patients are candidates for this intensive intervention. For those ineligible for transplant, novel targeted therapies and immunotherapies are urgently required. Recent advances with agents, such as brentuximab vedotin for CD30-positive PTCLs and checkpoint inhibitors for specific PTCL subtypes, have shown considerable potential (28). Integrating these novel agents into salvage regimens or as maintenance therapy post-transplant may further improve patient outcomes.

Furthermore, the heterogeneity of PTCLs presents significant challenges in developing standardized treatment protocols. Our study encompassed various PTCL subtypes, each exhibiting distinct biological characteristics and treatment sensitivities. Future research should aim to establish subtype-specific treatment strategies, potentially guided by molecular profiling and biomarker analysis. This personalized approach could optimize treatment selection and enhance outcomes across the spectrum of PTCLs.

This study has several limitations, including its retrospective design and relatively small sample size. We defined transplant eligibility based on an upper age limit of 65 years. Additional studies are required to address the salvage chemotherapy outcomes for those ineligible for transplant. The heterogeneity of PTCL subtypes and the absence of a control group also restrict definitive conclusions. Notably, only two patients were treated with an asparaginase-containing regimen, while the ENKT subtype represented 25 patients in our cohort. Our centers utilize ProMACE as the primary first-line chemotherapy for the ENKT subtype. Despite this, the conclusions remain relevant, as PTCL-NOS was the most prevalent subtype in our cohort, and no significant survival differences were observed with the ENKTCL subtype. Prospective, randomized studies are necessary to establish the optimal sequencing of novel agents, chemotherapy, and allo-HSCT in R/R PTCLs.

In conclusion, DL-ICE chemotherapy followed by allo-HSCT represents a feasible and potentially effective strategy for patients with R/R PTCLs. The achievement of long-term survival in a subset of patients underscores the importance of timely transplantation. Delaying transplantation in hopes of achieving a better chemotherapy response may result in patients becoming ineligible for transplant and consequently lead to poorer outcomes. Early consideration of allo-HSCT, even for patients with less than CR, may provide the best chance for cure in this challenging patient population. Future studies should focus on optimizing patient selection for allo-HSCT, exploring novel strategies to reduce post-transplant relapse and GVHD, and developing personalized treatment strategies based on PTCL subtype and molecular characteristics.

## Data Availability

The raw data supporting the conclusions of this article will be made available by the authors, without undue reservation.
